# Salvianolic acid A inhibits ferroptosis and protects against intracerebral hemorrhage

**DOI:** 10.1038/s41598-024-63277-4

**Published:** 2024-05-30

**Authors:** Yunpeng Shi, Dongdong Yan, Chengrui Nan, Zhimin Sun, Yayu Zhuo, Haoran Huo, Qianxu Jin, Hongshan Yan, Zongmao Zhao

**Affiliations:** 1 Department of Neurosurgery, The Second Hospital of Hebei Medical University, Shijiazhuang, 050000 Hebei China; 2 Department of Neurosurgery, Third Hospital of Shijiazhuang, Shijiazhuang, 050000 Hebei China; 3 Department of Neurosurgery, The Fourth Hospital of Hebei Medical University, Shijiazhuang, 050000 Hebei China

**Keywords:** Salvianolic acid A, Ferroptosis, Intracerebral hemorrhage, Akt/GSK-3β, NRF2, Primary cortical neurons, Network pharmacology, Molecular docking, Drug discovery, Neuroscience

## Abstract

Intracerebral hemorrhage (ICH) is a common cerebral vascular disease with high incidence, disability, and mortality. Ferroptosis is a regulated type of iron-dependent, non-apoptotic programmed cell death. There is increasing evidence that ferroptosis may lead to neuronal damage mediated by hemorrhagic stroke mediated neuronal damage. Salvianolic acid A (SAA) is a natural bioactive polyphenol compound extracted from salvia miltiorrhiza, which has anti-inflammatory, antioxidant, and antifibrosis activities. SAA is reported to be an iron chelator that inhibits lipid peroxidation and provides neuroprotective effects. However, whether SAA improves neuronal ferroptosis mediated by hemorrhagic stroke remains unclear. The study aims to evaluate the therapeutic effect of SAA on Ferroptosis mediated by Intracerebral hemorrhage and explore its potential mechanisms. We constructed in vivo and in vitro models of intracerebral hemorrhage in rats. Multiple methods were used to analyze the inhibitory effect of SAA on ferroptosis in both in vivo and in vitro models of intracerebral hemorrhage in rats. Then, network pharmacology is used to identify potential targets and mechanisms for SAA treatment of ICH. The SAA target ICH network combines SAA and ICH targets with protein–protein interactions (PPIs). Find the specific mechanism of SAA acting on ferroptosis through molecular docking and functional enrichment analysis. In rats, SAA (10 mg/kg in vivo and 50 μM in vitro, p < 0.05) alleviated dyskinesia and brain injury in the ICH model by inhibiting ferroptosis (p < 0.05). The molecular docking results and functional enrichment analyses suggested that AKT (V-akt murine thymoma viral oncogene homolog) could mediate the effect of SAA. NRF2 (Nuclear factor erythroid 2-related factor 2) was a potential target of SAA. Our further experiments showed that salvianolic acid A enhanced the Akt /GSK-3β/Nrf2 signaling pathway activation in vivo and in vitro. At the same time, SAA significantly expanded the expression of GPX4, XCT proteins, and the nuclear expression of Nrf2, while the AKT inhibitor SH-6 and the Nrf2 inhibitor ML385 could reduce them to some extent. Therefore, SAA effectively ameliorated ICH-mediated neuronal ferroptosis. Meanwhile, one of the critical mechanisms of SAA inhibiting ferroptosis was activating the Akt/GSK-3β/Nrf2 signaling pathway.

## Introduction

Intracerebral hemorrhage (ICH) is the most severe and least treatable form of stroke, affecting approximately 2 million people worldwide each year, often with a poor prognosis^1^. ICH has high morbidity and mortality rates^2^. Although significant progress has been made in the surgical removal of hematoma, the prognosis of intracerebral hemorrhage is still poor due to primary and secondary injuries^3^. Primary injury is physical injury, increased cranial pressure, and surrounding edema caused by extravasation of blood. In contrast, secondary injury is peripheral vascular neurotoxicity and cell death induced by hemoglobin, heme, iron, etc.^4^. Potential therapies to reduce neurotoxicity and cell death from clinical intracerebral hemorrhage are currently the focus of research in the treatment of secondary ICH injury.

Ferroptosis is an iron-dependent form of non-apoptotic programmed cell death and is a new therapeutic target for iron-related cell death^5^. There is considerable evidence that ferroptosis is closely related to pathophysiological processes in various cancers, stroke, traumatic brain injury, and neurodegenerative diseases^6^. Ferroptosis plays a vital role in the injury of ICH^7^. After ICH, lysed red blood cells can release a large amount of (Hb)/ HAEM, metabolized by microglia and macrophages to form Ferrous/Ferric iron. Thus, many Reactive oxygen species (ROS) and lipid peroxidation are generated around hematoma^8^. Neuronal cell death is achieved by lipid peroxidation^9^. Microglia release large amounts of ferrous iron and form highly toxic hydroxyl radicals through the Fenton reaction. They cause damage to cell membranes, proteins, and nucleic acids, eventually causing irreversible damage that disrupts cell function and leads to neuronal death^10^. However, our current research on how to treat neuronal ferroptosis after ICH is limited.

Ferrostatin-1 (FER-1) is one of the most widely used and recognized specific inhibitors of Ferroptosis. However, due to its instability in vivo experiments, FER-1 is still limited in the clinical transformation process^11^. Therefore, finding a more stable and effective ferroptosis inhibitor is the focus of current research. Salvianolic acid A (SAA) is the main active component extracted from the Salvia Militarize root. It has antioxidant, anti-inflammatory, anticancer, and neuroprotective biological activities^12–15^ and has been used to treat cerebrovascular diseases since ancient times. Previous studies have shown that the ability of SAA to scavenge free radicals and inhibit lipid peroxidation was evaluated^16^. A recent study has shown that SAA improves neurological deficits, intracerebral hemorrhage, blood–brain barrier disruption, and vascular endothelial dysfunction^17^. Previous studies have shown that SAA exhibits metal ion chelation and can reduce iron-induced lipid peroxidation and MDA production^18^. Based on earlier studies in each group, we hypothesized that SAA might ameliorate neurological dysfunction in ICH by inhibiting ferroptosis in brain tissue. A growing number of studies have shown that network pharmacology approaches can effectively map the target space of new natural products, thus expanding the parachutable reach of proteins associated with various complex diseases^19^. In network pharmacology, differences in action between different compounds can be revealed by creating a network of interactions between drugs and disease-related targets^20^. In our study, we will apply network pharmacology to investigate the targets and mechanisms of SAA in preventing and treating ICH.

Current studies have shown that Nrf2 activation mediated by Akt/ GSK3 β can reduce ferroptosis^21^. NRF2 plays a vital part in handling cellular antioxidant reactions, controlling the expression of stand-up to oxidation and ferroptosis qualities, and playing a crucial role in interceding lipid peroxidation and ferroptosis^22^. Recent studies have shown that SAA alleviates brain injury by promoting Nrf2 activation and nuclear translocation in a rat model of subarachnoid hemorrhage^23^.

Therefore, based on the description presented above, it is reasonable to speculate that SAA may have a protective effect on ferroptosis after ICH with the involvement of the Akt/GSK-3β/Nrf2 pathway through the pharmacologic docking network. Therefore, we evaluated SAA's anti-ferroptosis effect and molecular mechanism by establishing hemin-induced primary rat cortical neurons in vitro. We further confirmed this result in the ICH rat model. This trial aims to provide some new therapies for ICH ferroptosis.

## Materials and methods

### Primary cortical neuronal cultures

Female Sprague–Dawley rats (10–12 weeks of age) were anesthetized with isopropyl ether at 18 days of pregnancy, disinfected with 75% ethanol, disinfected by laparotomy, and then brain tissue was removed on ice. The bilateral cerebral cortex of fetal rats was isolated. The meninges were removed, transferred to 1 mL of Hank’s balanced salt solution on ice, chopped, and digested with trypsin (BD Biosciences, USA), and terminated 0 L with DMEM medium (Gibco, USA) medium containing 10% fetal bovine serum (Gibco, USA). Cells were inoculated in six-well plates coated with cell adherent Reagent (APPLYGEN, China). Neurobasal medium supplemented with B27(2%), l-glutamine (1%), and Mycoplasma (1%) prevention Reagent was maintained at 37 °C and 5% CO_2_. Supplant the medium every three days.

### In vitro ICH model and drug therapy

Hemin (50 μM; Sigma-Aldrich) simulated hemorrhagic stroke in cultured neurons. Erastin (2.5 μM, MedChemExpress, USA) and RSL3(1 μM, MedChemExpress, USA) simulated ferroptosis in cultured neurons. Control cells were treated with DMEM medium without FBS.

Salvianolic acid A (MCE, USA, Purity: 99.75%, C_26_H_22_O_10_) was treated with a series of concentrations (30–60 μM) containing Hemin (50 μM; Sigma-Aldrich) in a DMEM medium. Fer-1 (MedChemExpress, USA, Purity: 99.96%, C_15_H_22_N_2_O_2_) concentration was 1 μM. Cell viability was determined using Cell Counting Kit-8 (Sigma, St. Louis, MO, USA) according to the manufacturer’s instructions. SH-6 (0.5 μM; Sigma-Aldrich, 124009, USA) and ML385(10 μM, MedChemExpress, USA) were used to inhibit AKT and NRF2. Z-VAD- FMK (10 μM, MedChemExpress, USA), Necrosulfonamide (1 μM, MedChemExpress, USA).

### Assay of intracellular reactive oxygen species (ROS)

ROS Assay Kit -Highly Sensitive DCFH-DA Kit (Do Jindo, Japan) was used to detect intracellular ROS. After the medium was evacuated, cells in each group were washed twice with HBSS, added to a highly sensitive DCFH-DA and DAPI working Solution, and cultured in an incubator (37 ℃, 5% CO2) for 30 min. After removing the Working solution, HBSS was used for cleaning, and a fluorescence microscope (DMI3000B, Leica, Germany) was used for observation. Image J (Image J 1.4, NIH, USA) was used to quantitatively analyze intracellular ROS (area × intensity), and three fields were randomly selected for each group.

### Experimental groups and animals

Adult male Sprague–Dawley (SD) rats (10–12 weeks of age) were housed within indistinguishable conditions (room temperature at 25 °C, 12 h light–dark cycle) and permitted to get nourishment and water freely. The animals used in the experiment weighed between 250 and 300 g. A total of 96 SD rats were randomly divided into four groups: the SHAM operation group, intracerebral hemorrhage (ICH) group, ICH+ salvianolic acid A(SAA)group, and ICH+ ferrostatin-1 (Fer-1) group. All experimental procedures were approved by the Research Ethics Committee of the Second Hospital of Hebei Medical University and strictly followed National Institutes of Health guidelines for animal care and use.

### Establishment of intracerebral hemorrhage SD rat model

After anesthetization by intraperitoneal injection of 2% pentobarbital sodium (40 mg/kg), the rats were kept in the prone position in a stereotaxic frame (NEUROSTAR, Germany). Collagenase IV (Sigma-Aldrich, St. Louis, MO) was infused into the right striatum of rats to actuate ICH. Collagenase is a protein that can degrade collagen in the basal layer of the blood–brain barrier, ultimately leading to microvascular rupture near the injection site^24^. Collagenase IV (0.2U) was dissolved in 1 µL normal saline at a rate of 0.2 µL/min to induce intracerebral hemorrhage injury. The operation of the Sham group was infused with 1 µL of normal saline.

SAA (10 mg/kg) was injected intraperitoneally into the SAA group 2 h after collagenase IV injection, followed by IP administration once a day for five days. FER-1 group, FER-1 (2.5 μmol/kg, MCE, USA) was injected intraperitoneally once daily, as in the SAA group. SD rats were sacrificed five days after the operation to collect brain tissue. The rats were euthanized with 400 mg/kg pentobarbital sodium. The euthanasia and anesthesia methods were carried out in accordance with the guidelines of the American Veterinary Medical Association.

### Neurological deficit scores

The neural function injury induced by ICH was quantified by modified Longa score and Bederson score at 1, 3, and 5 days after the in vivo model was established.

According to the Longa scoring method, the rating scale was 0–4: 0 (indicating no neurological impairment), 1 (failing to extend the left front paw fully), 2 (circling left), 3 (descending to the left); 4 (no spontaneous walking and low level of consciousness). SD rats with a score of 0 had no neurological defects. SD rats with a score of 4 indicating severe brain damage were excluded from the study.

According to the Bederson scoring method, the rating scale was 0–5: 0 (no deficits), 1 (Flexion of the forelimb is present), 2 (Forelimb was flexed with lower resistance to lateral push), 3 (Circle the paralyzed side when free to move), 4 (Longitudinal rotation or accompanying seizures), 5 (no movement). The higher the Bederson score, the more severe the damage.

### Behavioral tests

The FPT (forelimb placement test) and the CTT (corner turn test) were used to quantify sensorimotor function.

In the tentacle-induced forelimb placement test (FPT), the rats were grabbed with their forelimbs freely extended. Each forelimb was tested independently by touching the corresponding tentacles to the edge of the corner of the table. The uninjured rats could quickly place their forelimbs on the worktable on the same side of the stimulated whiskers. Depending on the extent of the injury, the rat could place the contralateral forelimb on the stimulated whiskers on the workbench. Each mouse forelimb was tested ten times, and the appropriate forelimb was placed at the edge of the working surface to determine the proportion of trials in response to whiskers.

The corner turn test (CTT) allowed rats to enter a 30° corner. The animal could turn left or right to get out of the corner. As the rat turned, its choice of direction was recorded. This was re-hashed six times, and the rate of right turns was calculated.

### Brain water content evaluation

The intracerebral hemorrhage model was established in SD rats. 5 days later, brain tissue was extracted after anesthesia of SD rats (2% pentobarbital sodium, 40 mg/kg). The brain was instantly removed and separated into three parts: left hemisphere, right hemisphere, and cerebellum. Immediately in electronic analysis balance (BS210S, SARTORIUS, Germany), Brain samples were weighed to get wet weight (WW). The brain samples were then dried at 100 °C for 24 h to obtain dry weight (DW). Cerebral water content was determined as (WW−DW)/WW × 100%.

### BBB permeability

Evan’s Blue Dye (0.5%, 5 mL/kg) was slowly injected into the right femoral vein 1 h before the rats were sacrificed under anesthesia. After cardiac perfusion with ordinary cold saline, the brain was quickly decapitated, and the brain tissue around the hematoma was weighed, homogenized in 50% TCA (trichloroacetic acid), and centrifuged. The supernatant was mixed with TCA and incubated overnight at 4 °C. After centrifugation, the collected supernatant was evaluated at 620 nm.

### Analysis hematoma assessment

The SD rats were sacrificed 5 days after modeling for hematoma evaluation. One rat in each group was randomly selected, and the brain was quickly taken after 4% PFA (Solarbio, Beijing, China) perfusion and cut into 1 mm thick brain sections. The sections were then imaged using the Olympus stereomicroscope (SZX7), and each slice's amount of hematoma was recorded. The size of the hematoma was measured by Image Pro-Plus 6.0. The total hematoma volume was calculated by adding the hematoma areas in each section and multiplying by the section thickness^25^.

### Paraffin section of rat brain

Rats were infused with 4% formaldehyde through the heart, and brain tissue was quickly harvested. After being fixed in 4% PFA (4 °C) for 6 h, paraffin embedding was performed. The embedded brain tissue was cut into 5 μm sections and baked overnight at 60 ℃.

### Hematoxylin and eosin (HE) staining

Paraffin sections of rat brain tissues in each group were roasted at 60 ℃ for 1 h, dewaxed twice in xylene, and rehydrated with alcohol and gradient concentration distilled water of gradient concentration. The sections were hatched in hematoxylin for 5 min, washed with water for 15 min, and stained with eosin for 30 s. After the solution was rinsed with distilled water, gradient ethanol was dehydrated, and xylene was used to clear it—neutral branch seal. The sections were then observed and imaged employing a light microscope (DM200, Leica, Germany).

### Prussian blue staining

This Prussian blue stain showed iron deposits in the brain tissue surrounding the hematoma. First, paraffin-embedded sections were conventionally dewaxed with xylene and hydrated with gradient ethanol. It was then dyed with Prussian blue for 15 min. After rinsing with distilled water, an eosin dyeing solution was added for 15 s. Then, the brain sections were dehydrated by ethanol gradient and transparent by xylene. Finally, the neutral resin was used to seal the sheet. The sections were then observed and imaged employing a light microscope (DM200, Leica, Germany).

### Transmission electron microscope

Mitochondrial morphological characteristics were observed under transmission electron microscopy (HITACHI, Japan) to determine the effects of SAA and Fer-1 on the mitochondrial structure of rats 5 days after the induction of ICH induction.

### Fluoro-Jade C (FJC) staining

Denature-modifying neurons were detected using Fluoro-Jade C kit staining (TR-100-FJ, Biosensis, Australia). First, paraffin-embedded sections were conventionally dewaxed with xylene and hydrated with gradient ethanol. The sections were rinsed with distilled water and incubated with potassium permanganate for 15 min. The sections were then immersed in FJC and incubated with a DAPI solution. Finally, DPX was used to cover. The inverted fluorescent microscope (DMI3000B, Leica, Germany) was used for independent observers to perform three sectioned counts of each brain of FJC-positive neurons. Quantitative analysis was performed using Image J software (Image J 1.4, NIH, USA). Data were expressed as cell/mm by the average number of FJC-positive neurons within the field.

### Evaluation of ferroptosis

Ferroptosis was assessed by detecting iron content, MDA, and GSH levels in brain tissue surrounding rats' hematoma or primary cortical neurons. Approximately 1 × 10^6^ rat primary cortical neurons from each group and 10 mg brain tissue from around the hematoma after fresh pre-cooled PBS perfusion were used for the following tests. Iron content, MDA, and GSH levels in brain tissue or primary cortical neurons around the hematoma of rats were determined using the Iron Assay Kit (AB83366, ABCAM, USA), Lipid Peroxidation Assay Kit (AB118970, ABCAM, USA), and the GSH Assay Kit (Nanjingjianjien, China).

### Western blot

The cell nucleus and cytoplasm were separated using a MinuteTM cytoplasmic nuclear isolation kit (SC-003, Invent, USA). The extracted brain tissue and primary cortical neurons were lysed using RIPA lysis buffer (Beyotime, China). Protein concentrations were determined using a BCA kit (23225, Thermo Fisher Scientific, USA), and homogeneous protein samples were isolated by SDS-PAGE and transferred to PVDF membranes (Sigma-Aldrich, USA). Seal with 5% skim milk for 2 h. Subsequently, the membrane was incubated at 4 °C with primary antibodies overnight: XCT (AB175186, Abcam, USA), GPX4 (AB125066, Abcam, USA), GAPDH (ab181602, Abcam, USA), Histone H3 (ab1791, Abcam, USA), AKT (AF6261, Affinity Bioscience, China). Phospho—AKT (AF0016, Affinity Bioscience, China), GSK—3 beta (AF5016, Affinity Bioscience, China). Phospho—GSK—3 beta (AF2016, Affinity Bioscience, China), NRF2 (ab92946, Abcam, USA). The membrane was then incubated with the anti-rabbit lgG(H&L) (GOAT) antibody DyLigtTM 800 conjugated (1:1000, ROCKLAND, America) for 1 h. Visual analysis was performed utilizing the Odyssey Imaging System (Li-COR, USA). Image J software (1.4, NIH, USA) was used to quantify the density of the imprinted strip.

### Network pharmacology approaches

We first collected as many potential targets for Salvianolic acid A(SAA) as possible, and the following four databases were used: (1) ChEMBL (https://www.ebi.ac.uk/chembl), (2) The TCM Systems Pharmacology Database and Analysis Platform (TCMSP, http://lsp.nwu.edu.cn/tcmsp.php). (3) STITCH (http://stitch.embl.de/), (4) BATMAN-CM (http://bionet.ncpsb.org/batman-tcm).(5) TCMIP V2.0 (http://www.tcmip .cn/TCMIP/index.php).

Potential targets for intracerebral hemorrhage were collected from the following resources: (1) OMIM (http://www.omim.org/), (2) GeneCards (http://www.genecards.org/), (3) NCBI-Gene (https://www.ncbi.nlm.nih.gov/gene/),(4) DisGeNET (https://www.disgenet.org/home/).

The typical target could be obtained by intersecting the putative target of SAA with the ICH therapeutic target using the Venn diagram. Then STRING 11.0 (http://string-db.org/) was used to construct the protein–protein Interactions (PPIs) network of common targets. Perform GO and KEGG (http://www.kegg.jp/kegg/kegg1.html)^26–28^ signaling pathway enrichment analysis on target genes using the R language cluster Profiler package and visualize the GO/KEGG functional enrichment results of the obtained genes using the R ggplot package. Then, Cytoscape was used to analyze the Top 10 in network string ranked by Degree method.

### Molecular docking

To download ligands in MOL2 format and proteins in PDB format, we selected the PubChem database (http://pubchem.ncbi.nlm.nih.gov/) and RCSB protein data (http://www.rcsb.org/). Pymol software (https://pymol.org/2/; version 2.4.1) was used to conduct dehydration and separation of ligands. The crystal conducted was introduced to AutoDockTools to create a docking grid box for targets. Molecular dockings were achieved via AutoDock Vina. A lower Vina score, one of the outcomes of molecular docking, indicates a more stable binding affinity between protein and ligand. Pymol software was used to visualize the complexes of protein and compound.

### Experimental designs and drugs

The experimental designs and drug use are described in detail in Fig. 9

### Statistical analysis

Data were analyzed using GraphPad Prism 7, and graphs were generated. All summary data were expressed as mean ± SEM. Neurological scores were analyzed using the Kruskal-Walli’s test and the Dunn multiple comparison test. One-way ANOVA was used for the other values, followed by Tukey’s multiple comparison test. The P value < 0.05 was considered statistically significant.

### Ethical statements

All experimental procedures were approved by the Research Ethics Committee of the Second Hospital of Hebei Medical University and strictly followed National Institutes of Health guidelines for animal care and use. This study is reported following the ARRIVE guidelines (https://arriveguidelines.org).

## Result

### Neurons were incubated and identified in vitro

**Figure 1:**
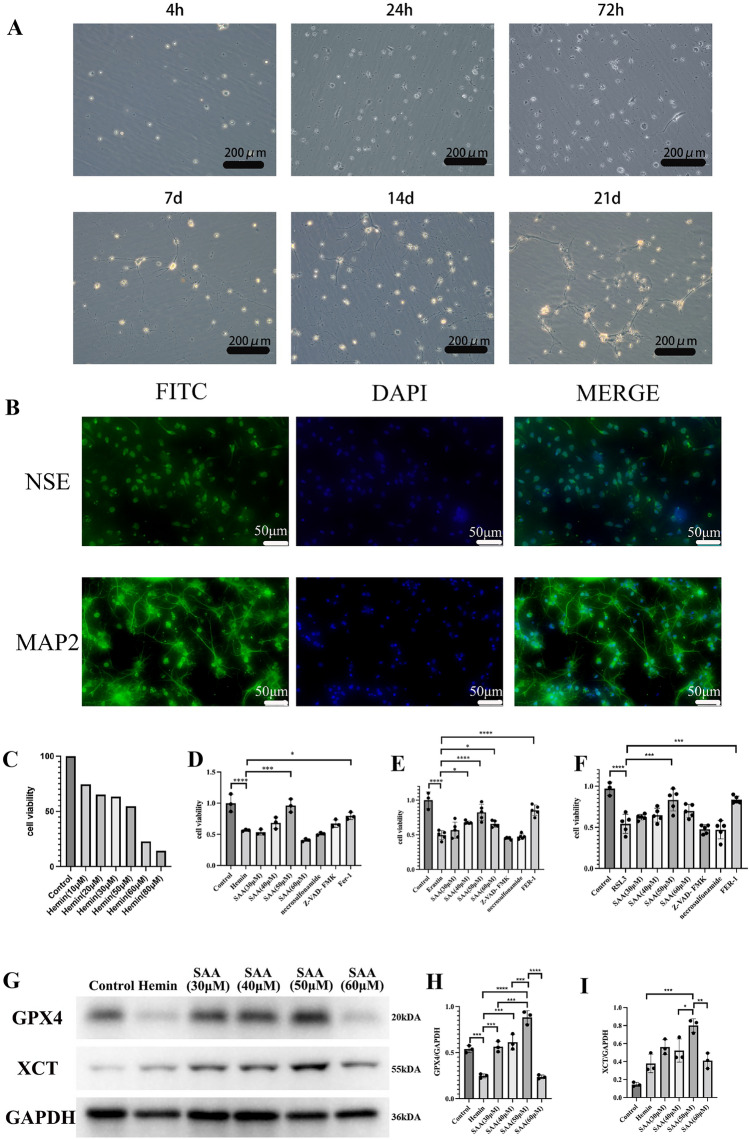
SAA can protect the cellular viability of primary cortical neurons during ferroptosis in vitro. (**A**) The inverted phase contrast microscope observed morphological changes in primary cortical neurons. (**B**) Primary cortical neurons were identified using immunofluorescence. (**C**) Cell viability was detected by CCK8, and the appropriate concentration of Hemin was selected to construct an in vitro model of intracerebral hemorrhage. (**D**–**F**) Primary cortical neurons were treated with Hemin, Erastin, or RSL3 in the absence or presence of SAA (30 μM,40 μM,50 μM,60 μM), necrosulfonamide (1 μM), Z-VAD-FMK (10 μM), or Fer-1 (1 μM) for 24 h, and then cell viability was measured. n = 3well/group. One-way ANOVA was used to compare multiple sets of data. ****P < 0.0001, ***P < 0.001, **P < 0.01, and *P < 0.05. n = 3/group.

To deeply analyze the relevant mechanisms of neuronal death and explore effective targets for preventing and treating intracerebral hemorrhage, we cultured cortical neuronal cells from rats in vitro. After 4 h, the neurons began to stick to the bottom of the culture flask under an inverted phase-contrast microscope. The cells were round, with an apparent refractive index, halo, and a few cells protruding from small protrusions. After 24 h, the number of protrusions and cell branches increased significantly. On day 7, a very dense network formed between the cells. On day 14, some cell protrusions shrank slightly. With the extension of the culture time, the growth of the cells gradually slowed down, and the cells continued to shrink, age, and gradually die. The cell survival time is about three weeks (Fig. 1A). The primary neuron cells were cultured for 14 days for immunofluorescence staining. Neurons stained positively for MAP2 and NSE (Fig. 1B). Therefore, in vitro cultured cells can be used for subsequent culture experiments.

### SAA can protect the cellular viability of primary cortical neurons during ferroptosis in vitro

Hemin was selected to simulate an in vitro model of intracerebral hemorrhage. CCK8 detected cell viability, and an appropriate concentration of Hemin was chosen to construct an in vitro model of intracerebral hemorrhage. The CCK8 results displayed that Hemin could reduce the cell viability of primary cortical neurons, and the concentration when the cell viability of primary cortical neurons was reduced to approximately 50% was used as the concentration to induce ICH (Fig. 1C). We then explored the effect of SAA on hemin-induced cytotoxicity of primary cortical neurons. The CCK8 results showed that SAA improved cell viability in a concentration-dependent way, as did FER-1, a specific selective inhibitor of ferroptosis (Fig. 1D). Erastin and RSL3 are currently recognized ferroptosis inducers as inhibitors of the cytosine/glutamate antiporter XCT and GPX4. We found that under certain circumstances, SAA can eliminate primary cortical cell death induced by Erastin and RSL3 in an in vitro model and Fer-1 (Fig. 1E and F).

Furthermore, Hemin-induced depletion of GPX4 led to a compensatory increase in the expression of the protective regulator xct downstream, while SAA treatment effectively enhanced XCT and GPX4(Fig. 1G–I). The results show that SAA (50 μM) could significantly increase XCT and GPX4 expression. Combined with the experimental results of CCK8, SAA (50uM) could dramatically improve cell viability when adding Hemin. Therefore, we chose SAA (50 μM) for the next in vitro experiments.

### SAA reduces intracellular ROS production

**Figure 2:**
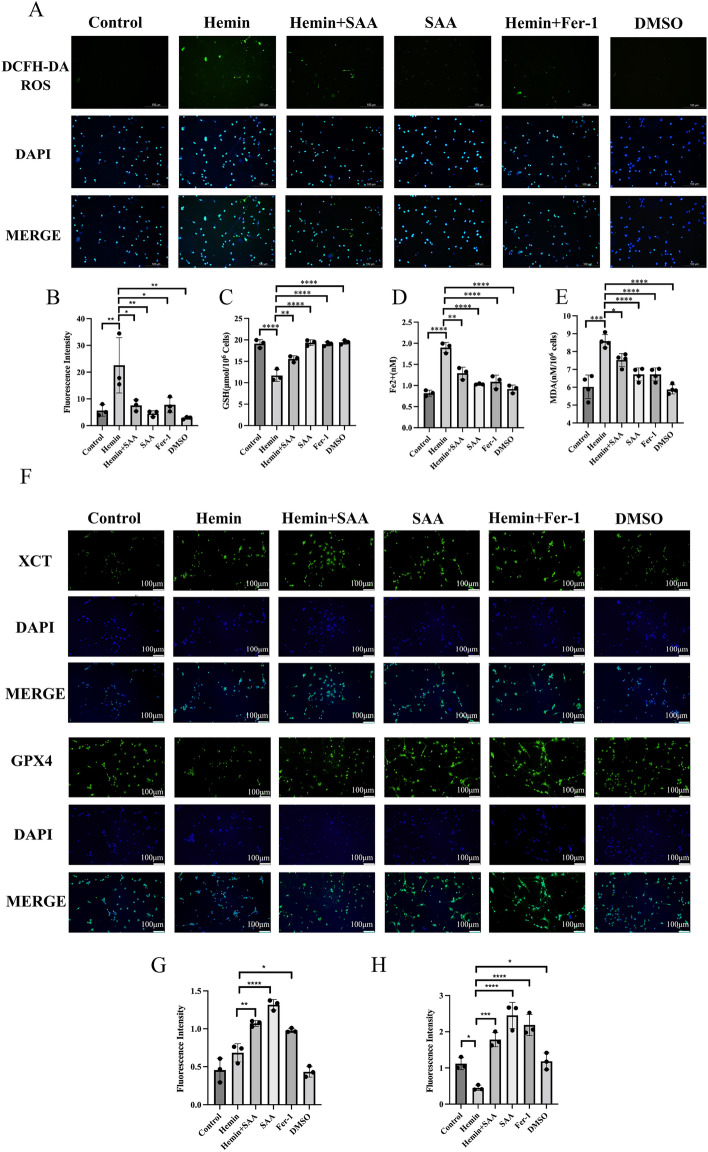
Effect of SAA on hemin-induced ferroptosis in primary cortical neurons. (**A** and **B**) Cellular ROS levels were evaluated by immunofluorescence staining using a DCFH-DA probe. (**C**–**E**) In the indicated primary cortical neurons, GSH levels, Fe2 + levels, and MDA levels were induced with Hemin for 24 h. (**F**) The expression of GPX4 and XCT was analyzed by immunofluorescence staining. (**G** and **H**) Relative fluorescence intensity statistics of the expression of GPX4 and XCT in different groups. n = 3/group. One-way ANOVA was used to compare multiple sets of data. ****P < 0.0001, ***P < 0.001, **P < 0.01, and *P < 0.05.

DCFH-DA is used to detect intracellular ROS levels. Green fluorescence is produced when ROS is produced in cells. The higher the fluorescence intensity, the higher the ROS level. As shown in Fig. 2A and Fig. 2B, the fluorescence intensity of the Hemin group was essentially higher than that of the control group. We discovered a significant decrease in fluorescence intensity in the SAA (50 μM) group, just as in the FER-1 group. This suggests that SAA (50 μM) mitigated the increase in intracellular ROS caused by Hemin to some extent. This inhibition of intracellular ROS production may reflect SAA’s ability to inhibit ferroptosis.

### SAA reduced MDA and Fe [2+] production and reversed the downregulation of GSH, XCT, and GPX4

Hemin-induced cortical neuronal cells showed significant ferroptosis, including intracellular MDA and Fe [2+] production, GSH and XCT, and GPX4 depletion. SAA (50 μM) or FER-1 reduced the increases induced by hemin in intracellular levels of MDA, Fe [2+], and GSH (Fig. 2C–E). Furthermore, SAA (50 μM) or FER-1 significantly increased XCT and GPX4 levels in cortical neurons (Fig. 2F–H). This may reflect SAA’s ability to inhibit ferroptosis.

### SAA mitigated ICH-induced brain injury in collagenase-induced rat models

**Figure 3:**
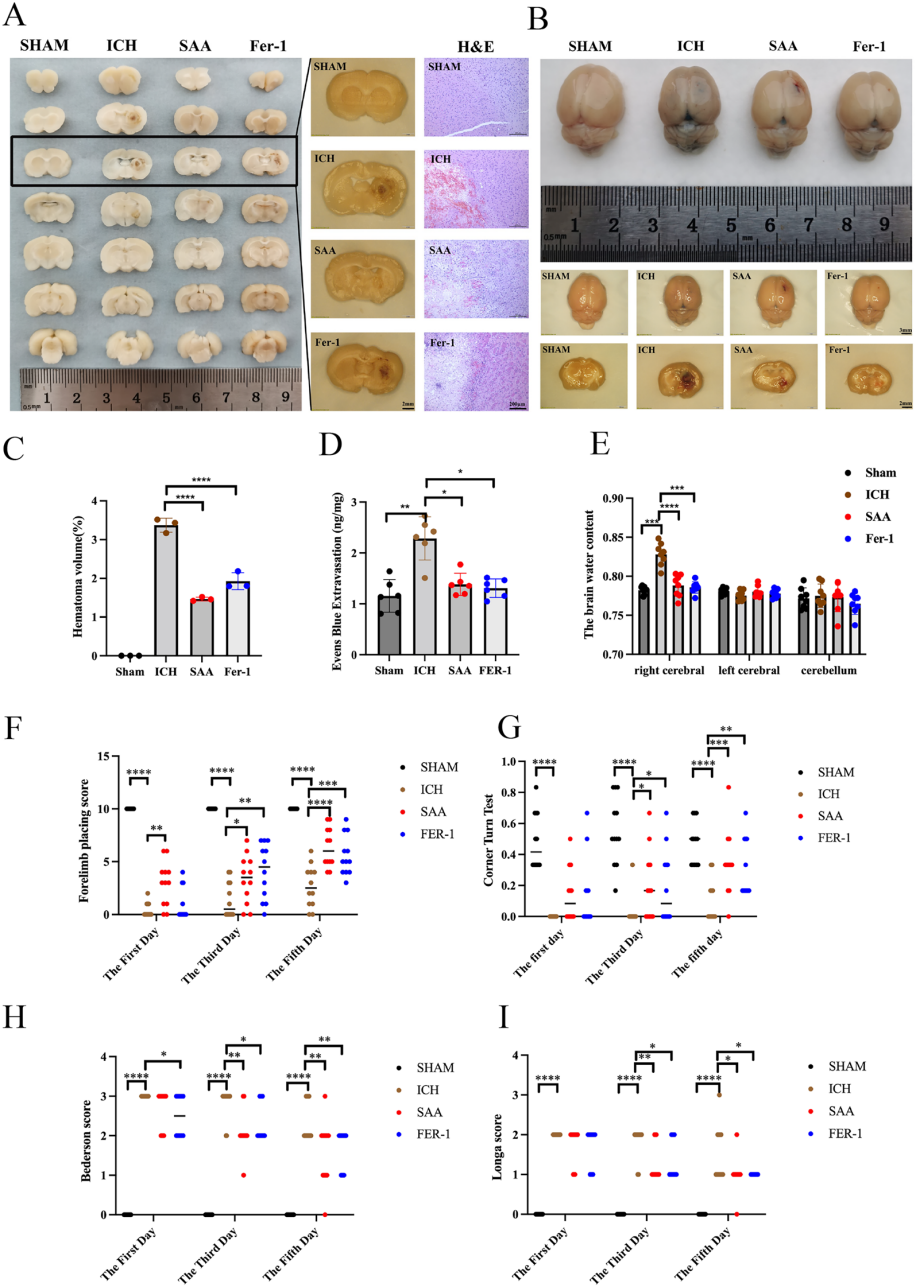
SAA mitigated ICH-induced brain injury in collagenase rat models. (**A**) Representative pictures of the hemorrhagic lesion and H&E staining in the Rats of different groups. (**B** and **D**) Representative pictures of the Evens blue extravasation of different groups. n = 6/group. (**C**) The percentage of hemorrhage volume to brain volume. n = 3/group (**E**) The cerebral water content of each group was examined to evaluate the effect of treatment on cerebral edema. n = 8/group (**F**) The forelimb placement test, n = 12/group (**G**) The corner turn test. n = 12/group. The (**H**) Longa and (**I**) Bedersen scores were performed to evaluate from day 1 to day 5 after ICH to assess recovery of neural function. n = 12/group. ****P < 0.0001, ***P < 0.001, **P < 0.01, and *P < 0.05.

Brain scans were performed to assess the efficacy of SAA in reducing the volume of the hematoma. As shown in Fig. 3A and Fig. 3C, the ICH group showed significant hematoma in brain tissue sections. Both the SAA and FER-1 groups significantly reduced the hematoma volume after ICH injury. HE dyes showed prominent edema and necrosis of brain tissue around the hematoma in the ICH group, irregular and loose arrangement of cells, enlarged extracellular space, and many infiltrations of inflammatory cell infiltration. However, pathological damage was significantly reduced in the SAA and FER-1 groups compared to the ICH groups. On the fifth day after ICH, the cerebral water content of each group was examined to evaluate the effect of treatment on cerebral edema. The brain water content of different anatomical brain structures showed that compared with the ICH group, the brain water content of the SAA group and FER-1 group was significantly reduced (Fig. 3E). Evan’s blue leaks were used to estimate the permeability of the blood–brain barrier. Figure 3B and D show that extravasation was significantly expanded within the ICH group compared to the SHAM group. SAA and FER-1 could facilitate the deterioration of extravasation.

### SAA alleviates ICH-induced neurological deficits in vivo

Neurobehavioral experiments were performed 1, 3, and 5 days after ICH to determine the effects of SAA and FER-1 on neurological deficits in ICH. Longa and Bedersen scores were used to assess neurological scores, and FPT and CTT were used to quantify sensorimotor function. Compared to the SHAM group, neurological scores in the ICH group decreased significantly 24 h after surgery and improved significantly after treatment with SAA and FER-1 (Fig. 3H and) I). At the same time, SAA and FER-1 significantly improved the correct position of the forelimb of ICH rats (Fig. 3F) and performed better in CTT (Fig. 3G).

### SAA reduces neurodegeneration and ferroptosis of brain tissue around the intracerebral hematoma

**Figure 4:**
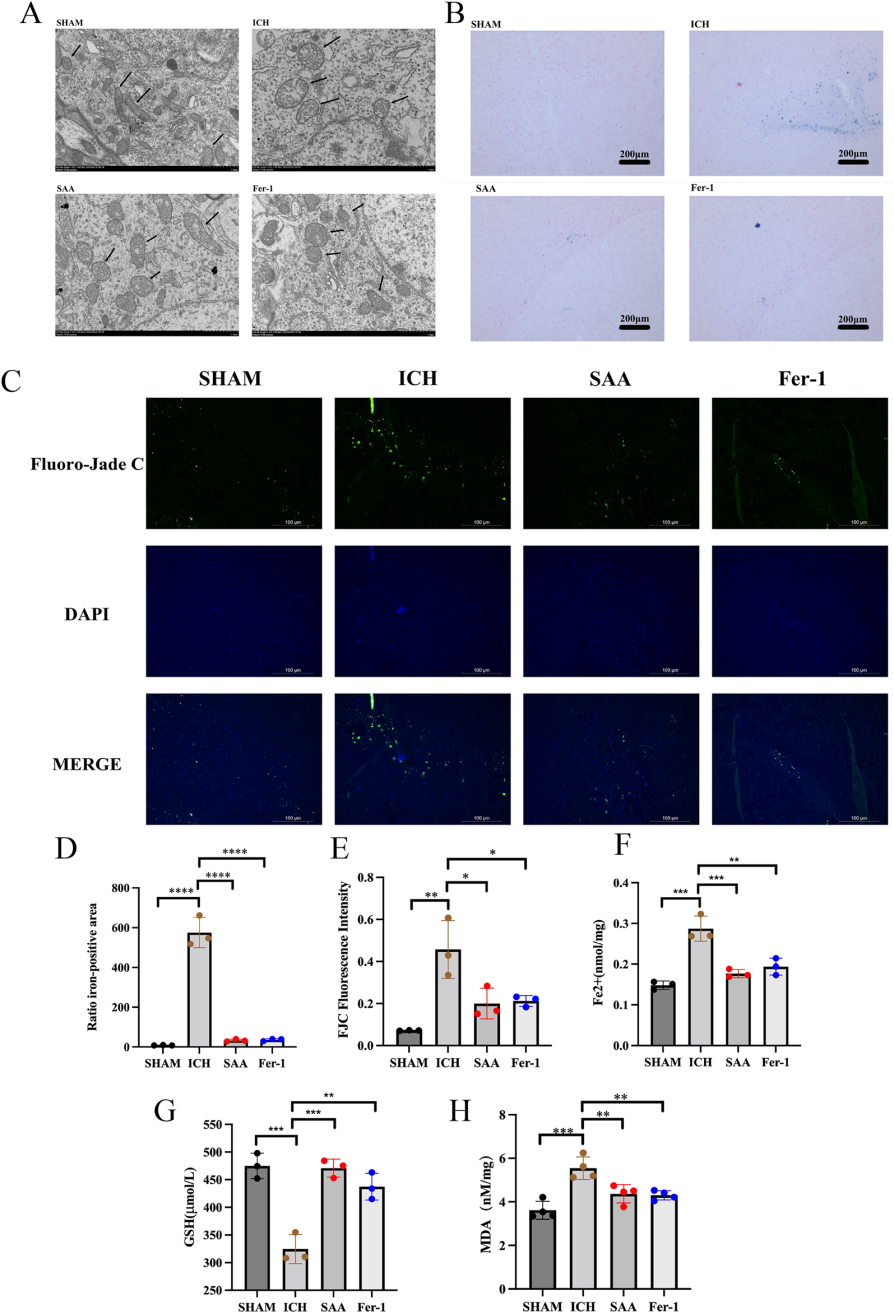
(**A**) Transmission electron microscopy was used to detect cells’ mitochondrial morphology in the SHAM, ICH, SAA, and Fer-1 groups. The black arrow indicates the mitochondria. (**B**–**D**) Representative images of Prussian blue staining and FJB staining of brain slices in rats of different groups. (**F**–**H**) GSH levels, Fe2+ levels, and MDA levels in intracerebral hemorrhage surrounding brain tissue were evaluated on day 5 after ICH. ****P < 0.0001, ***P < 0.001, **P < 0.01, and *P < 0.05. n = 3/group.

As ferroptosis would lead to morphological changes in mitochondria, transmission electron microscopy studies showed that SAA and FER-1 could significantly protect brain tissue damaged by ICH from mitochondrial crest loss and outer membrane rupture (Fig. 4A). The Fluoro-Jade C dyeing can locate the degeneration of the nerve cell, the dendritic, the axon, and the end. According to the Fluoro-Jade C staining, ICH damage caused significant neuronal degeneration in the brain tissue around the ICH rat blood. Treatment of SAA and Fer-1 can significantly reduce the number of FJC-positive cells (Fig. 4C and Fig. 4E). We used a Prussian blue dye to detect the position of iron in brain tissue around the hematoma.

Compared with the sham group, the deposit of the Fe [3+] in the peripheral brain tissue of the group was significantly increased. However, the treatment of SAA and Fer-1 has reversed the role (Fig. 4B and Fig. 4D). These findings indicate that SAA can effectively reduce iron deposits in brain tissue surrounding brain hemorrhage. The accumulation of DMA, GSH, and Fe [2+] was assessed to determine the effect of SAA on ferroptosis in the rat ICH model. The brain hemorrhage of rats contains higher concentrations of MDA, more Fe [2+], and fewer GSH. As we see in Fig. 4F–H, the MDA, GSH, and Fe [2+] improvement in the SAA and fern treatment groups is significantly improved relative to the group of groups. Treatment of SAA and Fer-1 can significantly improve MDA, GSH, and Fe [2+].

### Construction of the SAA-target-ICH network and Finding the Hub node

**Figure 5:**
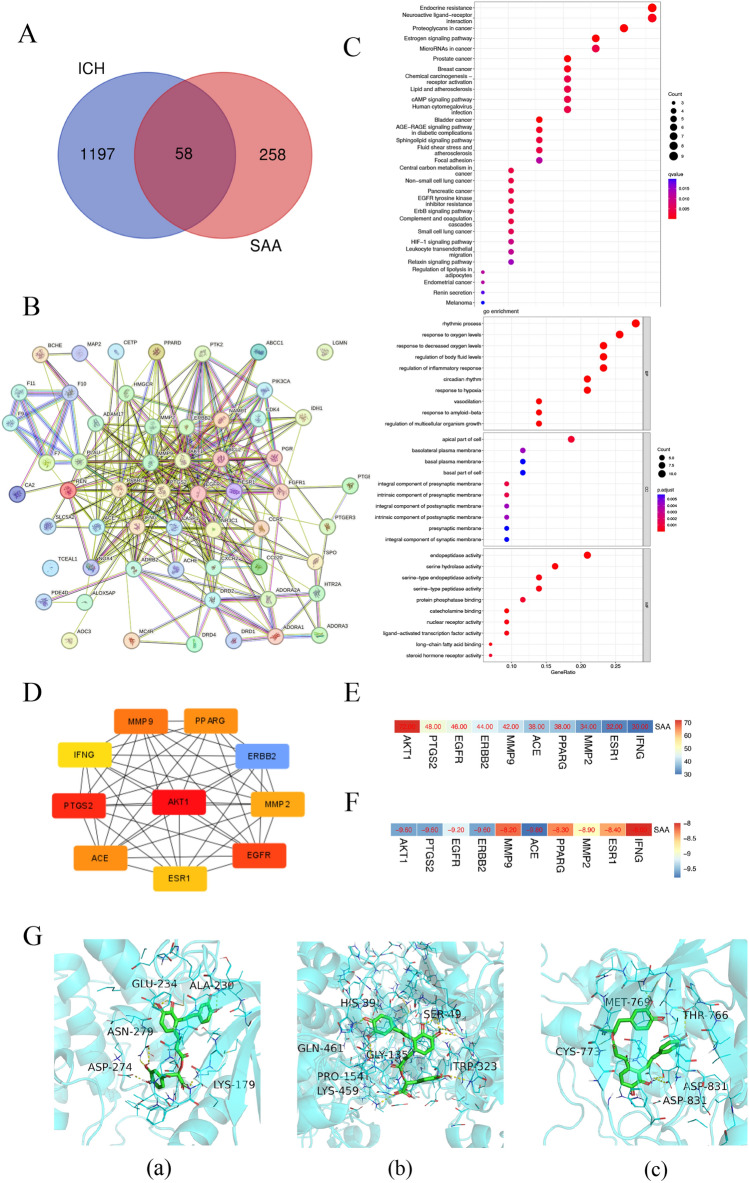
Construction of the SAA-target-ICH network and Find the Hub node. (**A**) Venn diagram showing the overlap of 58 genes between the potential targets of SAA and ICH-related genes. (**B**) PPI maps revealed the results of PPI network analysis. The larger node size indicates a higher number of protein interactions. (**C**) GO enrichment and KEGG pathway enrichment of DEGs. (**D**) The Cytohubba module in the Cytoscape software was used to find the HUB gene. The network interface diagram shows the connection of these HUB genes in the network. The darker the node color is, the higher the score is. (**E**) Heatmap of the top 10 in network string interactions ranked by degree method. (**F**) Heatmap of the docking scores of the active compounds of SAA and the target proteins. (**G**) Molecular docking results of “SAA compound-hub gene.” (**a**) SAA to AKT1; (**b**) SAA to PTGS2; (**c**) SAA to EGFR.

To illustrate the molecular mechanism of the impact of SAA on ICH, we constructed an SAA-targeted ICH network (Fig. 5). Potential SAA targets were searched through network pharmacology. There were 13 targets in TCMSP, 1 in Stitch and 1 in TCMIP, 4 in BATMAN and 334 in ChEMBL. After deleting redundant data, we found 316 potential SAA targets. After deleting redundant data, 1255 ICH-related genes could be obtained from OMIM, GEN-ECARDS, CBI-Gene, and DINET databases. 58 potential targets, including AKT, PIK3, P21, BCL2, NOX4, and EGFR, were identified, suggesting a possible protective role for SAA against ICH. A protein–protein interaction (PPIs) overlapping target network was constructed using TRING 11.0. The Cytohubba module in Cytoscape software was used to find the HUB gene. The network interface diagram shows the connection of these HUB genes in the network. The darker the node color is, the higher the score is. We found that AKT had the highest score and ranking. SAA probably acts on ICH through AKT. GO and KEGG analysis was performed through Matascape. The molecular functions of the target protein were mainly related to the circulatory system process, response to the rhythmic process, oxygen levels, and regulation of the inflammatory response. The pathways are primarily enriched in endocrine resistance, Neuroactive ligand − receptor interaction, and Proteoglycans in cancer.

### Molecular docking results

According to the results of the Cytohubba in Cytoscape software, we carried out molecular docking of the core target protein and SAA. Molecular docking between the top 10 target proteins (AKT1, PTGS2, EGFR, ERBB2, MMP9, ACE, PPARG, MMP2, ESR1, IFNG) and SAA was carried out using AutoDock Vina.

The docking scores of the strongest affinity of 10 core target proteins and SAA were visualized using the heatmap, as shown in Fig. 5F. The binding energy between target proteins and the active compounds was approximately between − 8.0 and − 9.8 kcal mol. Additionally, the target proteins have relatively vital docking energies, which means that SAA binds well to 10 core target proteins. Eventually, we chose the top 3 target protein macromolecules and SAA molecules with the best docking affinity for visualization with Pymol (Fig. 5G).

### SAA treatment significantly reduced ferroptosis in ICH rats through the Akt/GSK-3β/ Nrf2 pathway

**Figure 6:**
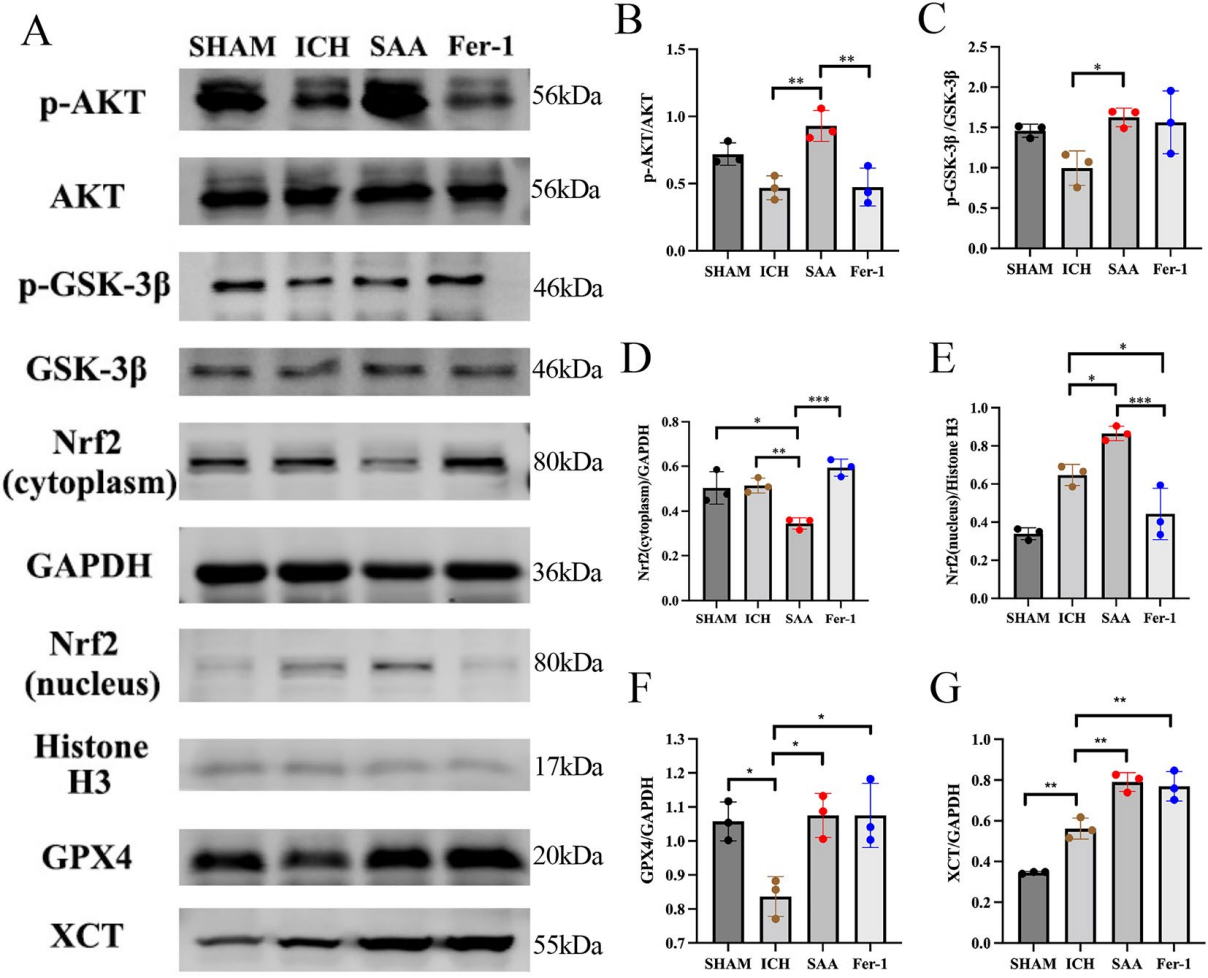
SAA treatment significantly reduced ferroptosis in ICH rats through the Akt/GSK-3β/ Nrf2 pathway. (**A**) Western blot analysis was used to detect the expression of phosphorylated and total Akt. Phosphorylated and total GSK-3β, Nrf2 proteins, GPX4 proteins, and XCT proteins. (**B**) Semiquantitative measurements of the ratio of phosphorylated to total Akt. (**C**) Semiquantitative measurements of the ratio of phosphorylated to total GSK-3β. (**D** and **E**) The protein expression levels of nuclear Nrf2 and cytosolic Nrf2 were analyzed by Western blot. (**F** and **H**) Protein expression levels of GPX4 and XCT were analyzed by Western blot. Values are expressed as means ± SEM. ****P < 0.0001, ***P < 0.001, **P < 0.01, and *P < 0.05. n = 3/group.

NRF2 is an essential regulator of ferroptosis. Vital downstream proteins include GPX4 and XCT. Through network pharmacology. AKT may be the target gene for SAA, and AKT and GSK-3β are upstream proteins of Nrf2. Therefore, we will investigate whether SAA up-regulates Nrf2 activity through the Akt/GSK-3β/ Nrf2 pathway. Their phosphorylation levels and the expression of GPX4 and XCT proteins were detected by Western blotting. As shown in Fig. 6, the phosphorylation levels of Akt, GSK-3β, and the translocation of nucleus Nrf2 significantly increase in the SAA group compared to group ICH. Similarly, XCT and GPX4 levels in group SAA increased considerably compared to group ICH.

### The Akt pathway is necessary for SAA to induce GSK-3β phosphorylation and Nrf2 nuclear translocation in primary cortical neurons

**Figure 7:**
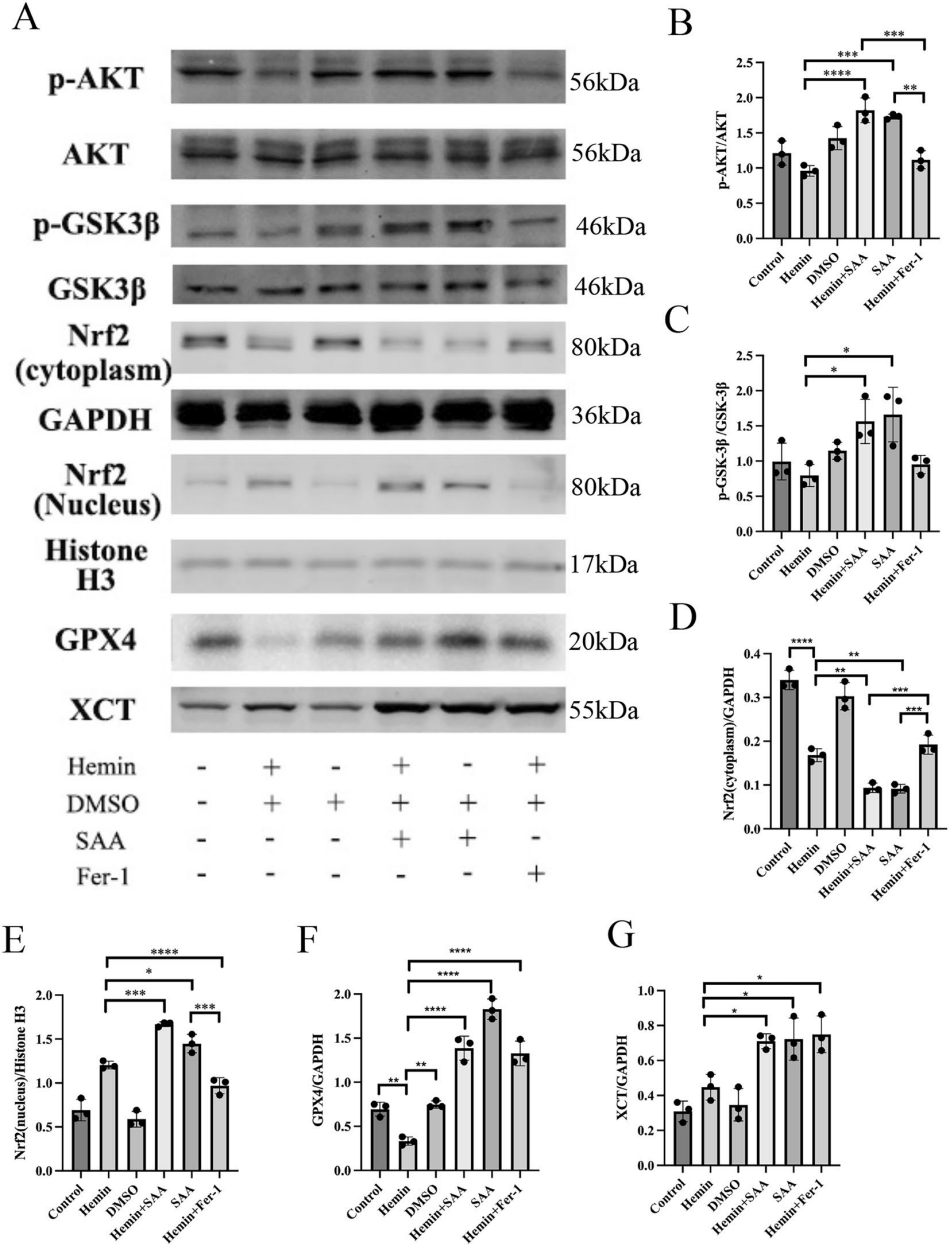
SAA treatment significantly reduced ferroptosis in primary cortical neurons through the Akt/GSK-3β/ Nrf2 pathway. (**A**) Western blot analysis was used to detect the expression of phosphorylated and total Akt. Phosphorylated and total GSK-3β, Nrf2 proteins, GPX4 proteins, and XCT proteins. (**B**) Semiquantitative measurements of the ratio of phosphorylated to total Akt. (**C**) Semiquantitative measurements of the ratio of phosphorylated to total GSK-3β. (**D** and **E**) The protein expression levels of nuclear Nrf2 and cytosolic Nrf2 were analyzed by Western blot. (**F** and **H**) Protein expression levels of GPX4 and XCT were analyzed by Western blot. Values are expressed as means ± SEM. ****P < 0.0001, ***P < 0.001, **P < 0.01, and *P < 0.05. n = 3/group.

**Figure 8:**
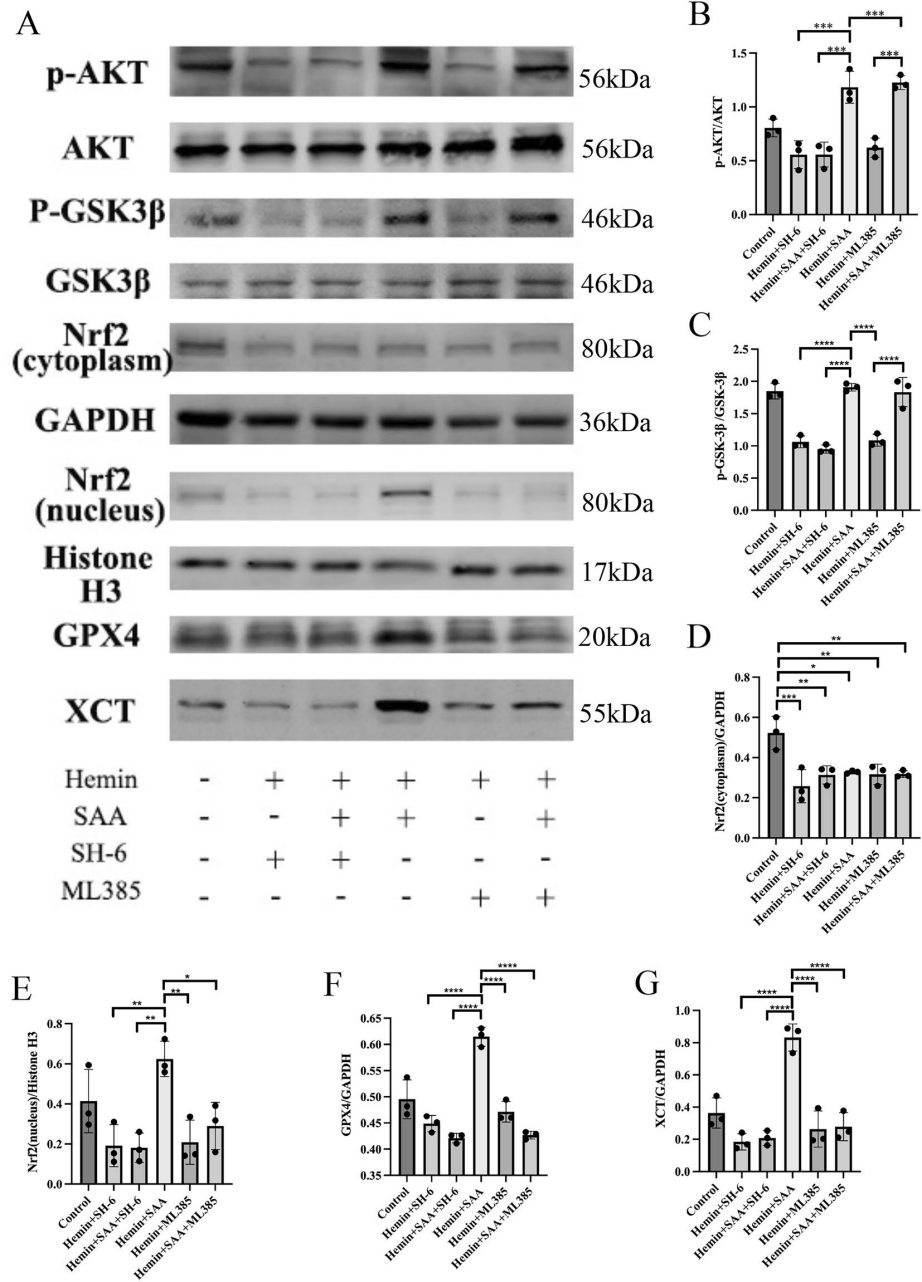
The Akt pathway is necessary for SAA to induce GSK-3β phosphorylation and Nrf2 nuclear translocation in primary cortical neurons. These effects were inhibited by treatment with SH-6 (an AKT inhibitor) and ML385 (an Nrf2 inhibitor). (**A**) Western blot analysis was used to detect the expression of phosphorylated and total Akt. Phosphorylated and total GSK-3β, Nrf2 proteins, GPX4 proteins, and XCT proteins. (**B**–**H**) Western blot analysis was used to detect the expression of p-AKT, GSK-3β, Nrf2, GPX4, and XCT proteins after treatment with the inhibitor of the Akt/GSK-3β/ Nrf2 pathway. Values are expressed as means ± SEM. Data were analyzed using a one-way analysis of variance, followed by Tukey’s multiple comparison test. ****P < 0.0001, ***P < 0.001, **P < 0.01, and *P < 0.05. n = 3/group.

As shown in Fig. 7, activation of Akt and GSK-3β and nuclear translocation of Nrf2 were mediated when primary cortical neurons were treated with SAA, which ultimately resulted in changes in XCT and GPX4 expression. FER-1, on the other hand, did not show this ability. Primary cortical neurons were treated with SAA with or without SH-6 and ML385 to determine whether SAA mediated the activation of Akt and GSK-3β and nuclear translocation of Nrf2. As shown in Fig. 8, SH-6 inhibited SAA-induced Akt, GSK-3β phosphorylation, Nrf2 nuclear expression, XCT, and GPX4 expression levels. ML385 inhibited Nrf2 nuclear expression, XCT, and GPX4 expression but had no significant effect on Akt and GSK-3β. These results suggest that SAA can inhibit ferroptosis by inducing AKT and GSK-3β phosphorylation and nuclear translocation of Nrf2.

### Experimental designs and drugs

**Figure 9:**
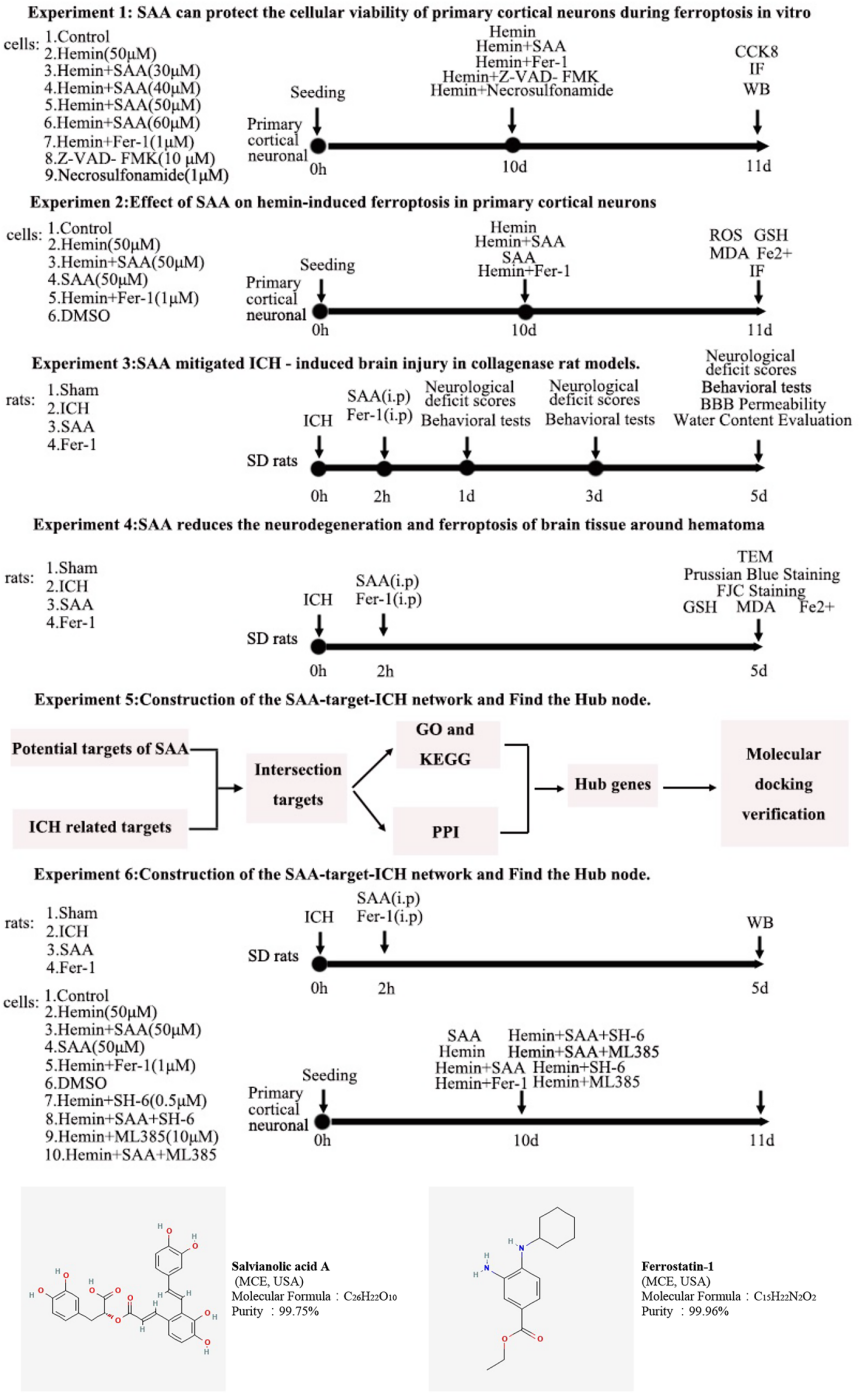
Experimental designs and drugs. SAA, salvianolic acid A; Fer-1, ferrostatin-1; ICH, intracerebral hemorrhage; IF, immunofluorescence; FJC, fluoro-Jade C; TEM, trans-on electron microscope; WB, western blot.

The experimental designs and drug use are described in detail in Fig. 9

## Discussion

Intracerebral hemorrhage is a fatal acute subtype of hemorrhagic stroke with a very high mortality rate. Within 1 month after the onset of ICH, only 60% of the patients survived^29^. The combination of primary and secondary injury from intracerebral hemorrhage causes this. Primary injury is physical injury, increased cranial pressure, and surrounding edema caused by extravasation of blood. In contrast, secondary injury is peripheral vascular neurotoxicity and cell death induced by hemoglobin, heme, and iron^4^. Unfortunately, although minimally invasive surgery for ICH has come a long way, ICH is still a headache that lacks specific treatment tools. Understanding the pathophysiology of a disease is always crucial to our search for novel diagnostic and therapeutic strategies. Emerging evidence supports the view that Hb/heme/iron is a significant factor in delayed cerebral edema and irreversible damage to neurons after ICH^30^. After ICH, the iron released by Hb in the blood produces a large amount of ROS, leading to nerve cell oxidative stress and secondary brain damage. Heme induces the production of many ROS and leads to cell damage by promoting lipid peroxidation^5^. Ferroptosis may play a vital role in this process. This would highlight ferroptosis as a promising target for future treatment of ICH.

Ferroptosis is a form of cell death regulated iron-dependent and produces large amounts of ROS^31^. The characteristic changes are driven by the production of lethal ROS produced by the Fenton reaction and the significant accumulation of lipid peroxides. The considerable iron accumulation provides rich ferrous iron for the Fenton reaction and leads to excessive hydroxides and hydroxyl radicals. The production of large amounts of ROS will damage nucleic acids, proteins, and cell membranes in cells, further aggravating the damage^10^. A large amount of Hb/heme/iron produced after ICH will induce ferroptosis. However, our current research on the classification of neuronal death after ICH and the related regulatory mechanism is still limited, so further neuronal ferroptosis after ICH has become a hot spot and complex in current research. It is urgent for us to find specific drugs to treat ICH to improve this situation.

SAA, a water-soluble phenolic acid isolated from the salvia miltiorrhiza root with significant antioxidant activity, can significantly improve cell viability in Hemin-induced primary cortical neurons in vitro. In addition, SAA greatly enhanced cell viability when Erastin and RSL3, two broad-spectrum ferroptosis inducers, were applied to primary cortical neurons. Previous studies have shown that SAA can significantly inhibit iron-induced MDA and intracellular ROS production and demonstrate its potential as an iron ion-chelating agent^18,32^. We set the DMSO group to examine the effect of the DMSO solvent on primary cortical neurons. Therefore, SAA is considered a potential treatment for ferroptosis associated with ICH. Current studies have shown that SAA can effectively treat stroke and ischemia/reperfusion (I/R) injury and has been an effective drug for treating cerebrovascular diseases since ancient times^17^. The pharmacokinetics of SAA indicate that SAA can act on the brain in large quantities and provide significant brain protection. Its metabolites show extra antibody lipid peroxidation's relatively high antioxidant activity^33^. SAA administration can significantly affect brain tissue, reduce brain edema after brain injury, improve nerve function defects, and improve anti-inflammatory and antioxidant capacity^34^. For in vivo experiments, the current research found that SAA (10 mg/kg) could significantly act on damaged brain tissue and substantially improve neurological deficits, intracerebral hemorrhage, and BBB disruption^17,35^. Therefore, we chose this concentration for in vivo experiments. A further in vivo experiment was carried out. SAA was administered intraperitoneally (10 mg/kg) 2 h after modeling, once a day for 6 consecutive days. The SAA-treated rats showed significant improvement in neurobehavior, with a significant therapeutic effect.

Meanwhile, ferric iron deposition in brain tissue around the hematoma was significantly reduced, and the degeneration of neurons caused by ICH injury was improved, showing a therapeutic effect on the ferroptosis of neurons after intracerebral hemorrhage. There is evidence that ferroptosis inhibitors can reduce cell damage after intracerebral hemorrhage in animal models of ICH, suggesting that ferroptosis inhibitors may be a potential drug for treating ICH^36^. Ferrostatin-1 (Fer-1), a ferroptosis inhibitor, can improve alarm protection and ameliorate acute intracerebral hemorrhage injury in the initial phase by alleviating ferroptosis. This potential mechanism prevents neuronal death by inhibiting ROS lipid production and reducing iron deposition^32^. Therefore, Fer-1 was used as a positive control for further experiments, and the potential regulatory mechanism of SAA regulating ferroptosis after ICH was further explored.

Many effective drugs do not act on a single target but play an essential role by regulating multiple proteins, so network pharmacology emerged. We identified 58 cross-targets by analyzing SAA-related and ICH-related targets. A protein–protein interaction network with 58 cross-targets was constructed, and 10 core targets from the network were identified. They are AKT1, EGFR, MMP9, REN, ERBB2, MMP2, DRD2, ADRB2, CR5, and CXCR2, among which AKT1 is the core target in the PPI network, indicating that it plays a crucial role in PPI network. We carried out molecular docking of the core target protein and SAA. A lower Vina score, one of the outcomes of molecular docking, indicates a more stable binding affinity between protein and ligand. The binding energy between target proteins and the active compounds was approximately between − 8.0 and − 9.8 kcal mol. Additionally, the target proteins AKT1 have relatively vital docking energies to SAA. Furthermore, the transcription factor NRF2 is a crucial regulator of cellular antioxidant response and regulation of ferroptosis, which plays a crucial role in neurological diseases^22^. NRF2 attenuates ferroptosis-mediated cell damage mainly by regulating SLC7A11 and GPX4^37,38^. SLC7A11 and SLC3A2 are integral in ferroptosis regulation and encode a cystine/glutamate antiporter xCT transporter^38^. Activation of Glutathione peroxidase 4 (GPX4) is the foundation of resistance to lipid peroxidation and Glutathione conversion. Our study showed that SAA ameliorated the hemin-induced decline in XCT and GPX4 in primary neurons. Among them, NRF2 is a key transcription factor that increases both simultaneously, and NRF2 is a critical downstream target of the hub gene AKT in the AKT/GSK-3b/Nrf2 pathway. Current studies have found that oxidative stress and neuronal apoptosis in ICH mice can be improved by the PI3K/Akt/Nrf2 signaling pathway^39^. It was found that SAA significantly increased the activities of glutathione peroxidase (GPx) and lowered the levels of malondialdehyde (MDA) and reactive oxygen species (ROS) by the Akt/GSK-3β/Nrf2 signaling pathway^40^. Activation of the Akt signaling pathway improves neurological function and alleviates neuronal death after ICH^41^.

Meanwhile, activation of the Akt/GSK-3 β/Nrf2 pathway is closely associated with reduced ferroptosis in cell models, inhibiting neuronal ferroptosis and promoting neuronal survival in the hippocampus^42,43^. Therefore, we chose the AKT/GSK-3b/Nrf2 pathway for further experiments in this study through network pharmacology^44^. SAA treatment in the ICH rat model showed increased expression of p-Akt, P-GSK-3 β, and Nrf2 nuclear internalization. The primary neuronal intracerebral hemorrhage model increased the expression of P-Akt, P-GSK-3 β, and Nrf2 nuclear internalization. This suggests that SAA activates the Akt/GSK-3 β/Nrf2 pathway. Similarly, we demonstrated that blocking the Akt/GSK-3 β/Nrf2 pathway significantly attenuated the hemin-induced activation enhancement and ferroptosis resistance of primary neurons induced by SAA. Therefore, we demonstrate that SAA regulates and inhibits neuronal ferroptosis after intracerebral hemorrhage by activating the Akt/GSK-3 β/Nrf2 pathway.

In previous experiments, especially in neuroscience, there has been a widespread presence of male bias. Many scientists have believed that female rodents have too many variable factors for many years, and their hormonal changes are tricky. A sudden hormone surge can affect their behaviors and disrupt research results^45^. Males still dominate animal studies^46^. Therefore, most cerebral hemorrhage models currently use male rats^41,47,48^. However, currently, this viewpoint is no longer credible^49^. Therefore, we will further test the effects of SAA on female rats in subsequent experiments and whether hormone changes affect the efficacy of SAA. At the same time, we will further increase the sample size of organizational results.

Our study demonstrates that salvianolic acid A improves ferroptosis in rats with intracerebral hemorrhage by promoting NRF2 activation via Akt /GSK-3β. Given the critical role of ferroptosis in intracranial hemorrhage, these results may reveal a novel SAA-BASED therapy for patients with ICH, and the promotion of NRF2 activation through the Akt /GSK-3β pathway may be developed as a promising treatment for ICH. Although this study provides therapeutic value for treating ICH, the experimental results and clinical application need further validation.

## Conclusions

SAA effectively ameliorated ICH-mediated neuronal ferroptosis. Meanwhile, one of the critical mechanisms of SAA inhibiting ferroptosis was activating the Akt/GSK-3β/Nrf2 signaling pathway.

## Data Availability

The data used to support the findings of this study are included in the article.
